# Comparison of analysis methods and design choices for treatment-by-period interaction in unidirectional switch designs: a simulation study

**DOI:** 10.1186/s12874-022-01765-9

**Published:** 2022-11-17

**Authors:** Zhuozhao Zhan, Geertruida H. de Bock, Edwin R. van den Heuvel

**Affiliations:** 1 Department of Mathematics and Computer Science, Eindhoven University of Technology, Eindhoven, The Netherlands; 2 Department of Epidemiology, University Medical Center Groningen, University of Groningen, Groningen, The Netherlands

**Keywords:** Stepped wedge design, Hybrid design, Clustered randomized controlled trial, Power analysis, Monte Carlo simulation

## Abstract

**Background:**

Due to identifiability problems, statistical inference about treatment-by-period interactions has not been discussed for stepped wedge designs in the literature thus far. Unidirectional switch designs (USDs) generalize the stepped wedge designs and allow for estimation and testing of treatment-by-period interaction in its many flexible design forms.

**Methods:**

Under different forms of the USDs, we simulated binary data at both aggregated and individual levels and studied the performances of the generalized linear mixed model (GLMM) and the marginal model with generalized estimation equations (GEE) for estimating and testing treatment-by-period interactions.

**Results:**

The parallel group design had the highest power for detecting the treatment-by-period interactions. While there was no substantial difference between aggregated-level and individual-level analysis, the GLMM had better point estimates than the marginal model with GEE. Furthermore, the optimal USD for estimating the average treatment effect was not efficient for treatment-by-period interaction and the marginal model with GEE required a substantial number of clusters to yield unbiased estimates of the interaction parameters when the correlation structure is autoregressive of order 1 (AR1). On the other hand, marginal model with GEE had better coverages than GLMM under the AR1 correlation structure.

**Conclusion:**

From the designs and methods evaluated, in general, parallel group design with a GLMM is, preferred for estimation and testing of treatment-by-period interaction in a clustered randomized controlled trial for a binary outcome.

**Supplementary Information:**

The online version contains supplementary material available at 10.1186/s12874-022-01765-9.

## Background

**Fig. 1:**
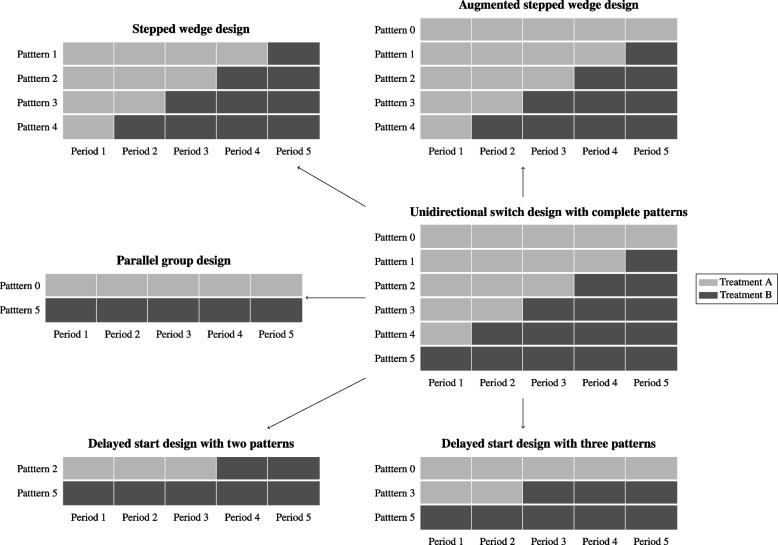
Schematic illustration of the USD design families

The family of unidirectional switch designs (USDs) [1, 2], which includes the parallel group design (PGD), stepped wedge design (SWD), and delayed start design (DSD) [3] and allows for unidirectional switches from a control to a new treatment, can be used as a randomized controlled trial for estimation of treatment effect. A “complete-pattern” USD with 5 time points is visualized in Fig. 1 (see also [3]). In general, different switching moments from control to intervention create different treatment patterns and the family of USD is a collection of designs that randomly allocate (group of) participants to some, but not necessarily all, patterns according to pre-specified probabilities. Different variations of USDs utilize different patterns. For instance, a PGD can be considered as a variation of USD which only uses the pure control and treatment pattern.

Efficiencies of different USD variations, in terms of estimation and hypothesis testing for the treatment effect, have been established in literature [2–5]. These results are all derived under the assumption of a constant treatment effect across the period.

Due to the sequential nature of treatment switching, violation of a homogeneous treatment effect may cause a substantial bias in the estimation when not addressed properly. For instance, if treatment had a higher effect on the outcome at a later period, then not taking into account such interaction may lead to an overestimated overall treatment effect since the treatment is less allocated at earlier periods than later periods. Therefore, it is important to investigate the presence of time-dependent treatment effects before making the assumption of a stationary treatment effect. This is usually addressed via hypothesis testing for the treatment-by-period interactions. However, so far little work has been published on this subject for the family of USDs (except for PGDs). Thus the main objective of this study is to investigate different analysis approaches for estimating and testing treatment-by-period interactions for USDs. Of course, not all variations of USD have identifiable treatment-by-period interaction effects (e.g., SWD) and different designs also lead to different efficiencies. Therefore, we study the analysis approaches for each variation of the USD that has estimable treatment-by-period interaction and compare them across different designs as well. Nevertheless, there might exist more complex forms of treatment-by-period interaction such as the time-on-treatment effect [6] where the treatment effect depends on the time since the onset of the intervention. In this study, we focus on the simple two-way interaction with no additional modifiers.

Furthermore, we focus on binary clinical outcomes when clusters of participants are being randomized (clustered randomized trial), and we assume that at each period a new set of participants from the cluster enters the trial (cross-sectional study). And we only consider “standard” designs that all clusters are being observed during the same (calender) periods [7]. We study the performances of different test statistics with simulations for different USDs. We compare statistical tests (Type III tests for the treatment-by-period interaction effects) from the generalized linear mixed model (GLMM) and from the marginal model with generalized estimating equations (GEE), both at aggregated and individual-level data.

The definition of the USD will be briefly explained in Section 2 and the estimation problem of the SWD for treatment-by-period interaction will be illustrated. We will list the USD variations that we will study with Monte-Carlo simulations. Furthermore, our simulation settings and the four analysis methods will be discussed and briefly reviewed in Section 2 as well. In Section 3, simulation results will be presented and discussed followed by a summary and discussion in the final section.

## Methods

Based on the idea of the stepped wedge design, switching treatments (from control to the treatment of interest) at different periods leads to different sequences of treatment patterns, or patterns in short (See Fig. 1). USDs refer to a family of designs that consists of all or a few of these patterns. The probabilities $p_0, p_1, \dots , p_{T}$ for each of the patterns are the probabilities of randomly allocating clusters or groups of participants to the corresponding patterns where the index represents the total number of treated periods in each pattern. By setting some of the probabilities to zero, certain patterns would be excluded from the trial. Note that we assume here (in line with literature on SWDs) that the clinical outcome is collected on each participant at the end of each period. The PGD is a USD with pure intervention and control patterns $p_0 + p_{T}=1$ (see Fig. 1). Although no switching is present in this design, it may be viewed as a special case of the USD where switching just happened before the trial started and after the trial ended for the pure treatment and control pattern, respectively. The commonly used form of SWDs is a USD with only “middle” patterns as illustrated in Fig. 1 ($p_0=p_{T}=0$). The DSD is either $p_{T} + p_s =1$, or $p_{T} + p_s + p_0=1$, where $s \in \{1,2,\dots ,T-1\}$. Girling and Hemming’s hybrid design [2] can also be considered as a special case of the complete pattern USD where $p_1=\cdots =p_{T-1}$ and $p_0=p_T >0$. Optimal choices for these probabilities for the estimation of the treatment effect (when no treatment-by-period interaction exists) has been addressed in literature [2, 3].

Identifiability of all treatment-by-period interaction effects requires participants with control and with treatment at each period. This is not the case for SWDs. To see why SWD is not identifiable, consider a SWD study with $T=4$ periods and $T-1=3$ switch moments as shown in Table 1, where the population average response at each cell is expressed in terms of combinations of a general mean $\mu _0$, the *j*th period effect $\beta _j$ for $j=1,\dots ,T$, an overall treatment effect $\theta _0$, and a period-specific treatment effect $\delta _j$ as a difference with respect to the overall treatment effect at period *j*. There are $2T - 2$ unique cells (out of $T^2-T$ cells) but $2T+1$ parameters are specified. Even if a certain period, say period *T*, is considered as the reference period which is common in most statistical software packages, and the corresponding parameter $\beta _T$ and $\delta _T$ is set to 0, there is still not enough degrees of freedom unless further restrictions are being made on the parameters. The way to mitigate the problem is to require designs to have both treatment arms present at all periods. In that setting, the number of unique cells is 2*T* (from $T^2 + T$ cells) and there will be $2T+2$ parameters. The restriction $\beta _T=\delta _T =0$, which are essential and common parameter constraints, solves the identifiability problem. Indeed, in this extended design, one additional parameter, namely $\delta _1$, is introduced but two additional unique cells are introduced as well, which compensates for the degrees of freedom deficiency. Note that Table 1 is more general than a specific parametric model with certain distributional assumptions for the outcome variables. For instance, considering an outcome $Y_{ijk}$ of subject $k, (k=1,\dots ,n)$ at period $j (j=1,\dots ,T)$ from switch pattern $i, (i=1,\dots ,T-1)$, any generalized linear (mixed) model with the following specification of the marginal mean $\mu _{ijk}=\mathbb {E}(Y_{ijk})$:

$$\begin{aligned} g(\mu _{ijk}) = \mu _0 + \beta _j + \theta _j \cdot x_{ij} \end{aligned}$$

where *g* is any monotonic mapping between the mean and the linear predictor, $\beta _j$ is the effect of the *j*th period, and $x_{ij}$ is a treatment indicator that equals 1 when treated and 0 otherwise with corresponding time-dependent treatment effect $\theta _j = \theta _0 + \delta _j$, conforms to the specification of Table 1. Such visualization might become convenient when investigating more complicated identifiability problems such as the case of three-way interactions or treatment-by-period interaction with a cluster-specific fixed effect.

**Table 1:** Visualization of the treatment-by-period interaction for a SWD

Switch	Period 1	Period 2	Period 3	Period 4
Switch 1	$\mu _0+\beta _1$	$\mu _0+\beta _2+\theta _0+\delta _2$	$\mu _0+\beta _3+\theta _0+\delta _3$	$\mu _0+\beta _4+\theta _0+\delta _4$
Switch 2	$\mu _0+\beta _1$	$\mu _0+\beta _2$	$\mu _0+\beta _3+\theta _0+\delta _3$	$\mu _0+\beta _4+\theta _0+\delta _4$
Switch 3	$\mu _0+\beta _1$	$\mu _0+\beta _2$	$\mu _0+\beta _3$	$\mu _0+\beta _4+\theta _0+\delta _4$

### Choices of USD

Since it is required to have both treatment arms present at each period, designs that satisfy this condition will be considered in the simulation. Such a condition suggests choosing designs with the pure intervention (or control) pattern combined with any other patterns. We will study the uniform PGD ($p_0=p_T =1/2$), uniform (3-pattern) DSD ($p_0 = p_s = p_T = 1/3$) (in short, DSDu), and the uniform (full-pattern) USD ($p_0=p_1=\cdots =p_T=1/(T+1)$) (USDu). We will also study the optimal DSD (DSDo), and the optimal USD optimized for the estimation of the overall treatment effect under the assumption of no treatment-by-period interactions for continuous outcomes. The probabilities $p_i$s are calculated according to  [3]. For the optimal DSD they are $p_0=(1-\rho + n\rho s)/[2(1-\rho +n\rho T]$, $p_s=n\rho T/[2(1-\rho + n\rho T)]$, and $p_T = [1-\rho + n\rho (T-s)]/[2(1-\rho +n\rho T)]$, where $\rho$ is the intraclass correlation coefficient, *n* the number of subjects per cluster per period, *T* the total number of periods, *s* the index of the “middle” switching pattern in DSD. For the optimal USD, the probabilities are given by $p_0 = p_T = (1-\rho + n\rho )/[2(1-\rho + n\rho T)]$, and $p_1 = \cdots =p_{T-1} = n\rho /(1-\rho + n\rho T)$. It should be noted that these optimal designs are developed for continuous outcomes with the assumption of a large number of clusters. For binary outcomes, the optimal designs are unknown and we expect the aforementioned optimal designs to remain approximately optimal for large sample sizes. Furthermore, $\rho$ for the binary outcome is calculated differently as detailed in Section 2.3. An additional complication, in the simulation study with a finite number of clusters, is the allocation of the clusters according to the exact optimal probabilities will lead to fractional numbers of clusters. We consider 2 different rounding options for the optimal USD. Following the advice of [3], we first consider rounding options for the pure control and treatment pattern, this leads to two options (up or down). Rounding up the optimal proportions yields one unique USD (denoted as USDo1), while several options are possible when we round down. After rounding down patterns 0 and 5, we need to either round up patterns 1 and 4 or round up patterns 2 and 3 (options leading to non-symmetric allocations are excluded). Since the asymptotic relative efficiencies for estimating the treatment effect is negligible between the two options [3], we decide to choose the former option (denoted as UDSo2), since we’d like to put more clusters to patterns that are closer to the pure treatment and control patterns. The numbers of clusters allocated to each pattern for all design candidates are listed in Table 2 (Section 3).

**﻿Table 2:** Type I error and power of GLMM and GEE at individual and aggregated level for testing treatment-by-period interactions with exchangeable correlation structure ($N_h$ denotes the number of clusters allocated to switch pattern *h*)

Design	$\sigma _c^2$	Cluster allocation						Type I error				Power			
		$N_0$	$N_1$	$N_2$	$N_3$	$N_4$	$N_5$	GLMM Agg	GEE Agg	GLMM Ind	GEE Ind	GLMM Agg	GEE Agg	GLMM Ind	GEE Ind
PGD	0.2	24	0	0	0	0	24	0.053	0.050	0.052	0.050	0.994	0.981	0.994	0.981
PGD	0.5	24	0	0	0	0	24	0.040	0.044	0.039	0.042	0.992	0.917	0.991	0.914
PGD	0.8	24	0	0	0	0	24	0.060	0.056	0.060	0.055	0.984	0.842	0.984	0.829
DSDu	0.2	16	0	0	16	0	16	0.043	0.040	0.043	0.039	0.962	0.861	0.961	0.858
DSDu	0.5	16	0	0	16	0	16	0.040	0.037	0.039	0.037	0.948	0.712	0.948	0.704
DSDu	0.8	16	0	0	16	0	16	0.046	0.051	0.046	0.051	0.951	0.606	0.951	0.588
DSDo	0.2	14	0	0	24	0	10	0.049	0.035	0.050	0.034	0.913	0.724	0.911	0.716
DSDo	0.5	14	0	0	24	0	10	0.042	0.024	0.041	0.022	0.893	0.557	0.893	0.543
DSDo	0.8	14	0	0	24	0	10	0.043	0.037	0.042	0.038	0.857	0.451	0.854	0.445
USDu	0.2	8	8	8	8	8	8	0.047	0.036	0.047	0.036	0.858	0.659	0.858	0.653
USDu	0.5	8	8	8	8	8	8	0.032	0.037	0.032	0.037	0.841	0.511	0.840	0.510
USDu	0.8	8	8	8	8	8	8	0.059	0.046	0.058	0.046	0.827	0.405	0.825	0.402
USDo(1)	0.2	6	9	9	9	9	6	0.039	0.034	0.038	0.034	0.777	0.487	0.775	0.481
USDo(1)	0.5	6	9	9	9	9	6	0.032	0.022	0.031	0.022	0.737	0.365	0.733	0.363
USDo(1)	0.8	6	9	9	9	9	6	0.035	0.030	0.034	0.031	0.696	0.295	0.619	0.289
USDo(2)	0.2	5	10	9	9	10	5	0.036	0.029	0.036	0.029	0.700	0.415	0.698	0.408
USDo(2)	0.5	5	10	9	9	10	5	0.035	0.021	0.035	0.022	0.676	0.320	0.674	0.314
USDo(2)	0.8	5	10	9	9	10	5	0.036	0.034	0.034	0.035	0.644	0.255	0.642	0.251

### Brief review of analysis methods

Statistical analysis of USDs can be broken down into two general approaches. Either the data can be analysed at an aggregated level by taking summary measures at cluster-period combinations, or the data can be analysed at an individual level. The aggregated measure is denoted by $Y_{ij} \equiv M(Y_{ij1},\cdots ,Y_{ijn})$ with $M(\cdot )$, for instance, the average or sum, where $Y_{ijk}$ denotes the *k*th observation in cluster *i* of period *j*. The two candidate methods considered in this study can be applied either on an aggregated measure or on the individual outcomes. In the first method, namely the marginal model with GEE [8], variations between clusters are treated as nuisances and the cluster functions as the analysis unit with repeated observations (individuals) and both period and treatment enter the model as fixed effects. The focus of the analysis is on the fixed effects averaged over the clusters [9]. The second analysis method is a subject-specific mixed effects model with clusters as random effect and period and treatment again as fixed effects [2, 10]. Generally speaking, aggregation of the outcome does not preserve the within-cluster correlation structure for binary/binomial outcomes with a nonlinear link function. This is especially true for the working correlation of GEE. For instance, suppose the working correlation matrix at the individual level follows the Toeplitz correlation structure. This correlation structure is no longer true at the aggregated level. Reassuringly, this is less of a concern because the covariance is considered as nuisance parameters in GEE. Nevertheless, discussions on the relative merits of the two methods for estimation of a common treatment effect (without treatment-by-period interactions) are well-discussed in literature [11–14]. In general, the marginal model does not rely on the assumption of the correlation structure and is robust against misspecification. However, when the number of clusters is small, the empirical “sandwich” estimator [15–17] used in the model underestimates the true (co-)variances of the parameters and the Wald-type test is subject to inflated Type I error. On the other hand, the mixed effects model approach is sensitive to the specification of the covariance structure and the coefficients are more difficult to interpret on a population level.

### Simulation settings

A cross-sectional clustered randomized controlled trial that consists of *M* clusters ($M\in \{48, 192\}$^1^) in total and observed over $T = 5$ periods with subjects per period per cluster set to $n=50$. The outcome of interests $Y_{ijk}$ is assumed to be a Bernoulli random variable for subject $k\, (k=1,\dots , n)$ at period $j\, (j=1,\dots , T)$ from cluster $i\, (i=1,\dots , M)$. The following GLMM is used to generate the outcome $Y_{ijk}$:

1 $$\begin{aligned} \mathbb {E}(Y_{ijk}| b_{0i}) = \Phi (\mu _0 + b_{0i} + \beta _{j} + \theta _j x_{ij}), \end{aligned}$$

where $\Phi$ is the cumulative distribution function of the standard normal distribution, $\mu _0$ is a general intercept, $b_{0i} {\mathop {\sim }\limits ^{iid}} \mathcal {N}(0, \sigma _c^2)$ a random effect of cluster *i*, $\beta _{j}$ is the fixed effect of period *j* ($\beta _j=0.2 (j-3)$), $\theta _j = \theta _0 + \delta _j$ the period-specific treatment effect at period *j* and with $\theta _0 = -0.4$ the average treatment effect over periods. The $\delta _j$ is the difference with the average treatment effect at period *j*, two sets of $\delta _j$ is considered, namely a linear effect ($\delta _j = -0.1 (j-3)$) and a symmetric effect ($\delta _j=-0.1 |j-3|$). Finally, $x_{ij}$ is the treatment indicator for cluster *i* at period *j* taking the value of 1 if the cluster is allocated to the intervention, and 0 otherwise. In addition, a set of three different values $\{0.2, 0.5, 0.8\}$ is chosen for $\sigma _c^2$ and the ICC $\rho$ used to calculated each optimal designs is derived as

$$\begin{aligned} \rho = \frac{\sigma _c^2}{\sigma _c^2 + 1}. \end{aligned}$$

Besides the exchangeable correlation structure used in the aforementioned data generating process, we also considered a simulation setting with an autoregressive order 1 (AR1) correlation structure between time points at the cluster level. That is, the random effect $b_{0i}$ of cluster *i* is now replaced by a random effect $b_{ij}$. This random term is assumed to be multivariate normal with mean 0 and variance equal to $\sigma _c^2$ but now with additional correlation between two time points *j* and *k* as ${\text {corr}}(b_{ij},b_{ik}) = r^{|j-k|}$. Here, the correlation parameter is set to $r \in \{0.5,0.8\}$. Only aggregated-level analysis will be considered for AR1 since the correlation matrix at the individual level is cumbersome to specify and the true correlation structure is usually unknown in practice. Even at the aggregated level, the AR1 correlation structure specified at the latent variable level is not preserved at the outcome level and an AR1 working correlation structure will still be a misspecification for the true correlation structure. We thus only used exchangeable working correlation for GEE in this setting.

In addition, for all the simulation settings, the parameters are specified according to a subject-specific model. To translate these regression coefficients or parameters (denoted by $\varvec{\theta }_S$) into marginal model parameters (denoted by $\varvec{\theta }_M$), the following relationship is used:

$$\begin{aligned} \varvec{\theta }_M = \frac{\varvec{\theta }_S}{\sqrt{1+\sigma _c^2}}. \end{aligned}$$

Note that this relationship is exact since we used the probit link function $\Phi$ (i.e., the cumulative distribution function of the standard normal distribution) in combination with a normally distributed random effect for clusters in the simulation process  [18].

The same GLMM model (1) was used to fit each simulated data at the individual level. For the marginal model with GEE, the following marginal mean structure was considered:

$$\begin{aligned} \mathbb {E}(Y_{ijk}) = \Phi (\mu _M + \beta _{Mj} + \theta _{Mj}x_{ij}), \end{aligned}$$

where $\mu _M$, $\beta _{Mj}$, $\theta _{Mj}$ are the aforementioned marginal model parameters counterparts to the subject-specific model parameters $\mu _0$, $\beta _{0j}$, and $\theta _j$, respectively. Furthermore, an exchangeable working correlation structure was specified. On the other hand, for the aggregated analysis, the number of events $Y_{ij} = \sum \nolimits _{k=1}^n Y_{ijk}$ per cluster and period was summarized first, and a similar GLMM model

$$\begin{aligned} \mathbb {E}(Y_{ij}/n | b_{0i}) = \Phi (\mu _0 + b_{0i} + \beta _j + \theta _j x_{ij}) \end{aligned}$$

was used. Similarly, a marginal model with GEE was used to fit the cluster-period event counts.

Bias and coverage probabilities were summarized based on 1000 simulations. All simulations were conducted in SAS 9.4. For the marginal model with GEE, PROC GENMOD was used and coverage probabilities were based on the Wald-type confidence intervals. For GLMM, PROC GLIMMIX was used with Laplace approximation of the likelihood for estimating the parameters. The coverage probabilities were derived based on the Wald-type confidence intervals (asymptotically equivalent to the confidence interval based on t-distribution with the denominator degrees of freedom assumed to be infinite). Powers of the hypothesis test for the treatment-by-period interactions were calculated by the generalized score test based on empirical standard errors for the marginal model with GEE and based on the $\chi ^2$ test for GLMM. Type I errors were calculated under the simulation setting of $\delta _j=0$ for $j=1, 2,\dots , 5$. The default parameterization in SAS is to take the last period as reference, we changed this to the third period, since we have chosen $\beta _3=0$ and $\delta _3=0$. This means that the intercept $\mu _0$ has the interpretation of the event probability at period 3. For other variables, we followed the default parametrization in SAS. We also calculated the Monte-Carlo standard error (MCSE) for the simulation results.

## Results

To be economical with the presentation, most of the simulation results are deferred to the Additional files (Additional files 1, 2, 3 and 4). In this section, we will show some important findings.

### Bias and coverage probability

**Fig. 2:**
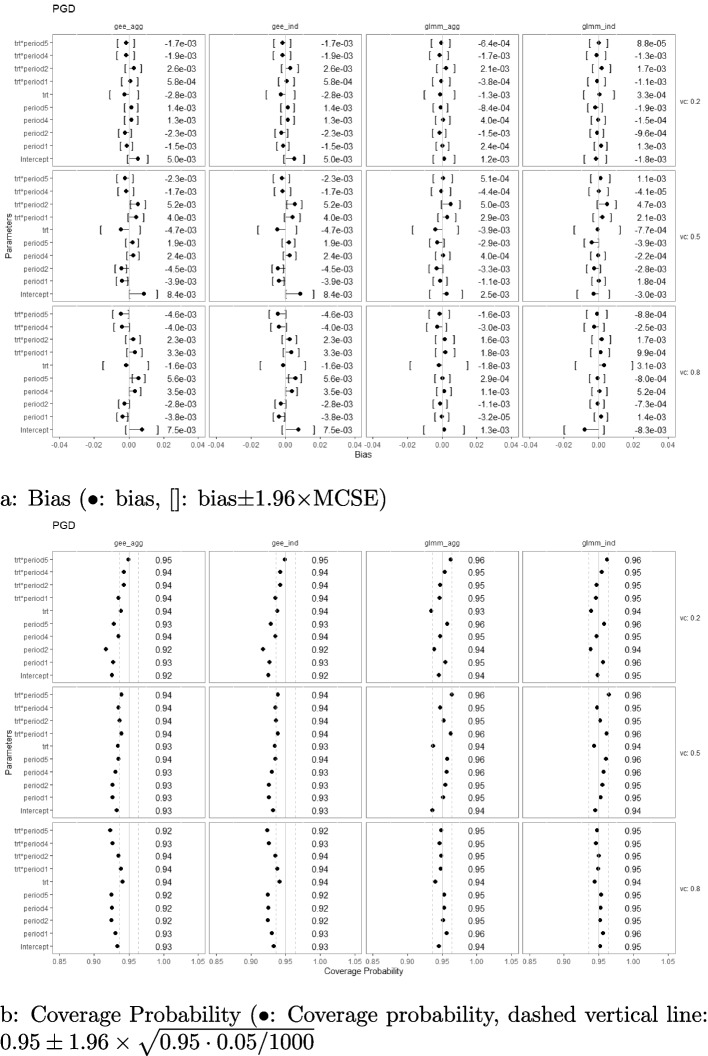
Estimation biases and coverage probabilities of the four approaches (GLMM at individual level (glmm_ind), GLMM at aggregated level (glmm_agg), marginal model with GEE at individual level (gee_ind), marginal model with GEE at aggregated level (gee_agg)) using the PGD under the settings of $M=48$, $n=50$, linear effect $\delta _j=-0.1(j-3)$, and $\sigma _c^2 \in \{0.2, 0.5, 0.8\}$ with an exchangeable correlation structure. (vc: variance component, trt*period1-5: treatment-by-period interaction at period 1-5)

**Fig. 3:**
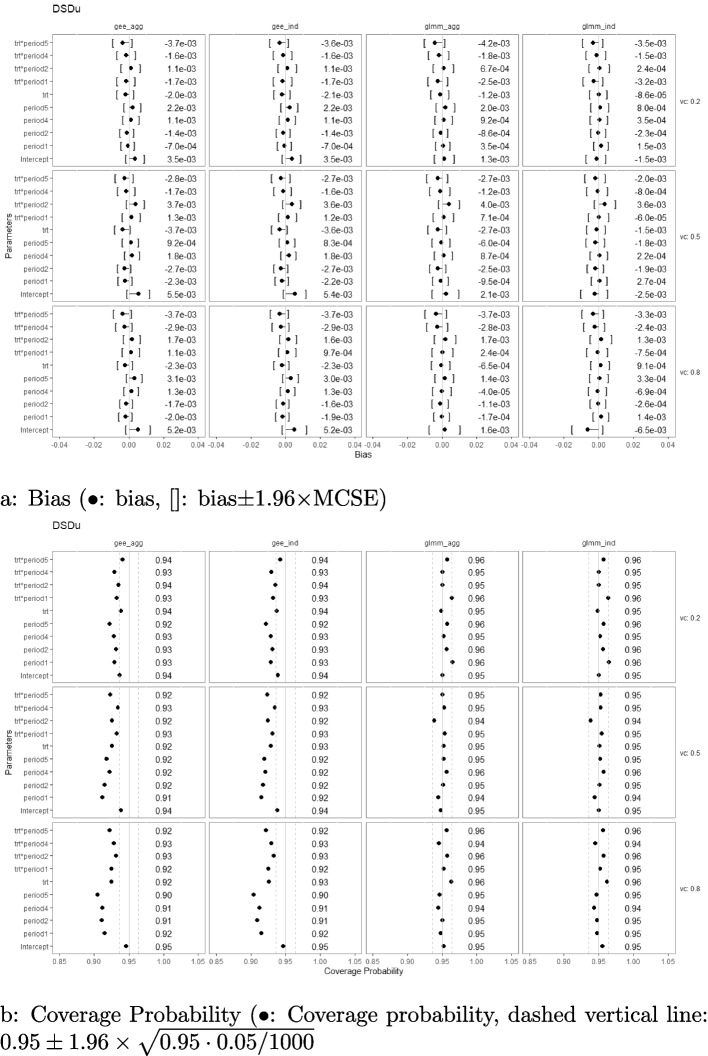
Estimation biases and coverage probabilities of the four approaches (GLMM at individual level (glmm_ind), GLMM at aggregated level (glmm_agg), marginal model with GEE at individual level (gee_ind), marginal model with GEE at aggregated level (gee_agg)) using the DSDu under the settings of $M=48$, $n=50$, linear effect $\delta _j=-0.1(j-3)$, and $\sigma _c^2 \in \{0.2, 0.5, 0.8\}$ with an exchangeable correlation structure. (vc: variance component, trt*period1-5: treatment-by-period interaction at period 1-5)

**Fig. 4:**
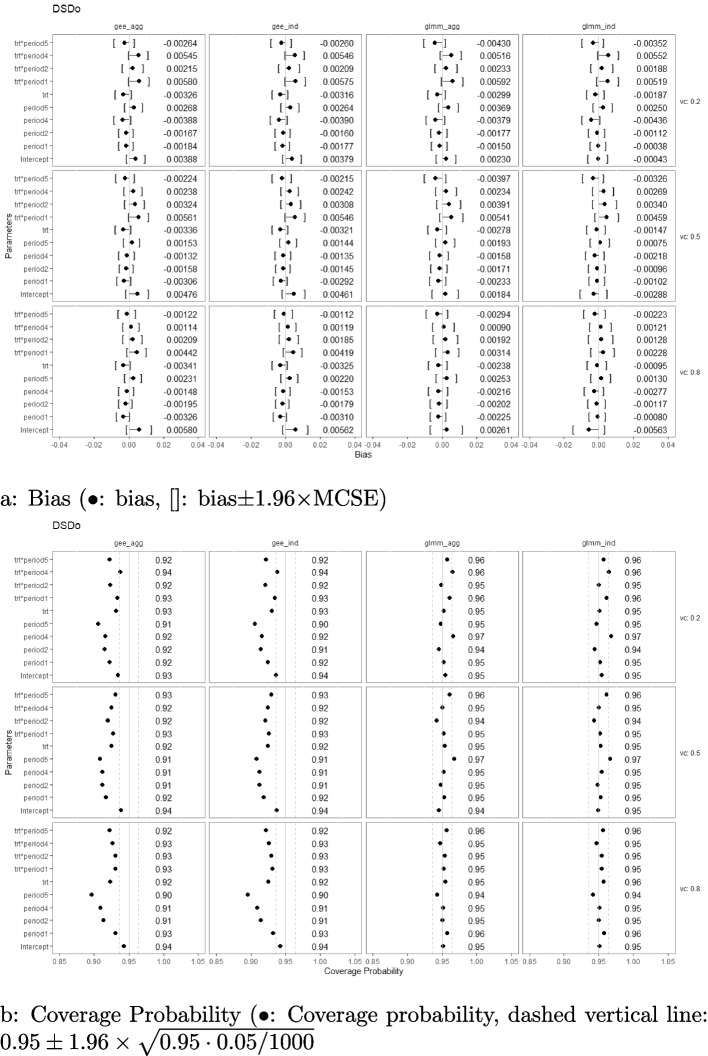
Estimation biases and coverage probabilities of the four approaches (GLMM at individual level (glmm_ind), GLMM at aggregated level (glmm_agg), marginal model with GEE at individual level (gee_ind), marginal model with GEE at aggregated level (gee_agg)) using the DSDo under the settings of $M=48$, $n=50$, linear effect $\delta _j=-0.1(j-3)$, and $\sigma _c^2 \in \{0.2, 0.5, 0.8\}$ with an exchangeable correlation structure. (vc: variance component, trt*period1-5: treatment-by-period interaction at period 1-5)

**Fig. 5:**
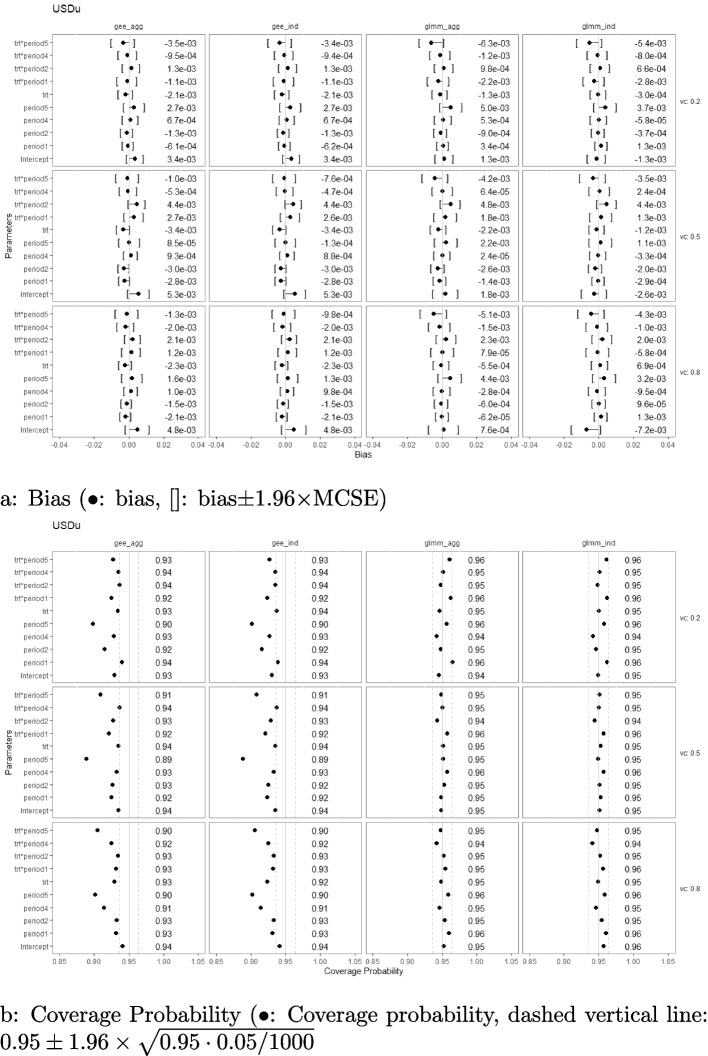
Estimation biases and coverage probabilities of the four approaches (GLMM at individual level (glmm_ind), GLMM at aggregated level (glmm_agg), marginal model with GEE at individual level (gee_ind), marginal model with GEE at aggregated level (gee_agg)) using the USDu under the settings of $M=48$, $n=50$, linear effect $\delta _j=-0.1(j-3)$, and $\sigma _c^2 \in \{0.2, 0.5, 0.8\}$ with an exchangeable correlation structure. (vc: variance component, trt*period1-5: treatment-by-period interaction at period 1-5)

**Fig. 6:**
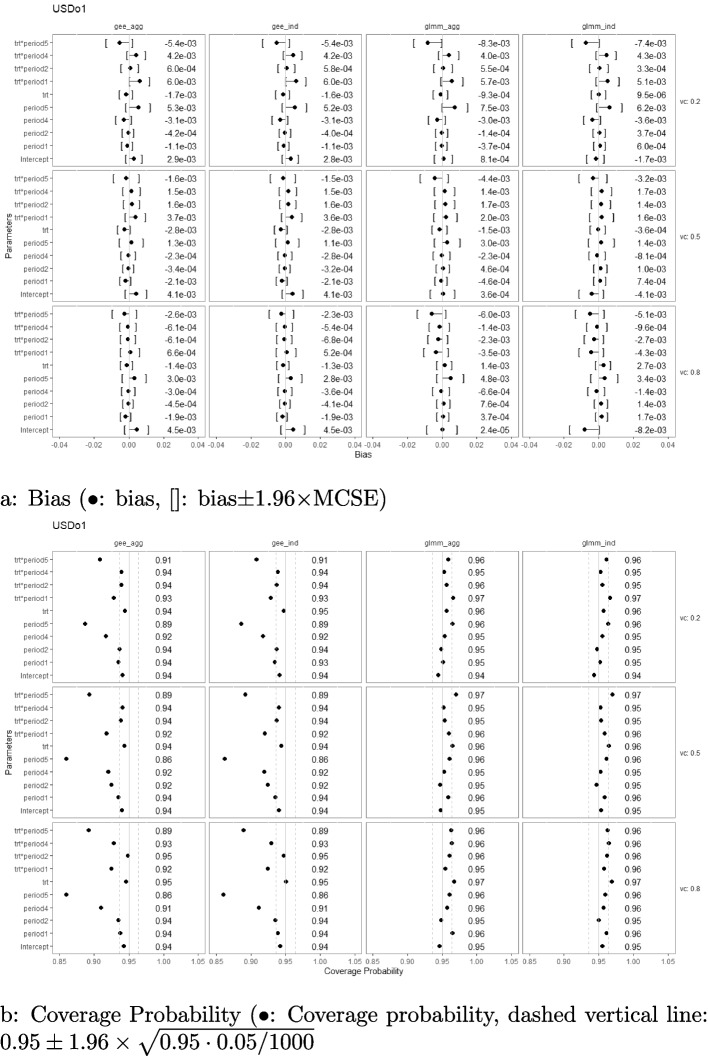
Estimation biases and coverage probabilities of the four approaches (GLMM at individual level (glmm_ind), GLMM at aggregated level (glmm_agg), marginal model with GEE at individual level (gee_ind), marginal model with GEE at aggregated level (gee_agg)) using the USDo1 under the settings of $M=48$, $n=50$, linear effect $\delta _j=-0.1(j-3)$, and $\sigma _c^2 \in \{0.2, 0.5, 0.8\}$ with an exchangeable correlation structure. (vc: variance component, trt*period1-5: treatment-by-period interaction at period 1-5)

**Fig. 7:**
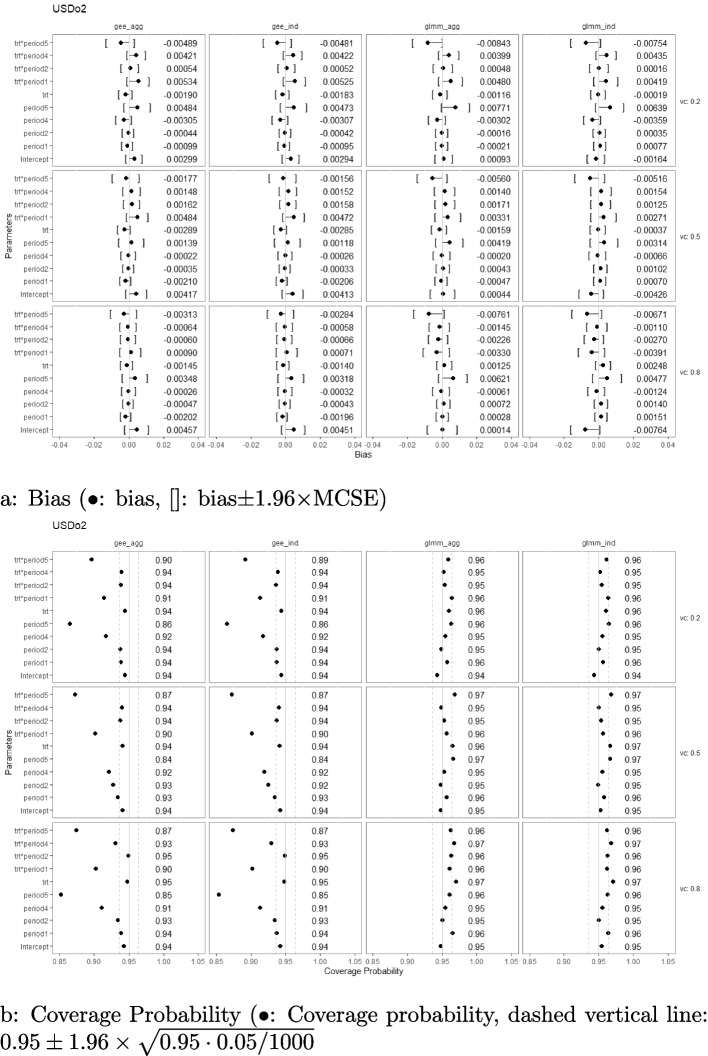
Estimation biases and coverage probabilities of the four approaches (GLMM at individual level (glmm_ind), GLMM at aggregated level (glmm_agg), marginal model with GEE at individual level (gee_ind), marginal model with GEE at aggregated level (gee_agg)) using the USDo2 under the settings of $M=48$, $n=50$, linear effect $\delta _j=-0.1(j-3)$, and $\sigma _c^2 \in \{0.2, 0.5, 0.8\}$ with an exchangeable correlation structure. (vc: variance component, trt*period1-5: treatment-by-period interaction at period 1-5)

Simulation results with regard to the bias and coverage probability are presented in Figs. 2, 3, 4, 5, 6, and 7 under the settings of $M=48$, linear interaction effect $\delta _j=-0.1(j-3)$, and $\sigma _c^2 \in \{0.2, 0.5, 0.8\}$ with an exchangeable correlation structure. All four analysis methods estimate the model parameters without bias across all USD designs. Furthermore, GLMM had close-to-nominal coverage probabilities at both aggregated and individual levels, while the marginal model with GEE had lower coverage probability among all designs at both levels. The coverages were especially poor for GEE when the estimand was the period effect and the treatment-by-period effect at period 5. Such phenomenon was even more severe in designs with extreme unbalanced treatment-control ratios at the first and last period (namely, USDo1 and USDo2). Note that Wald’s confidence interval used with GEE is well-known to fall under the nominal level in general and it is further lowered by unbalanced samples (unequal proportions of treatment and control within the period) [19].

**Fig. 8:**
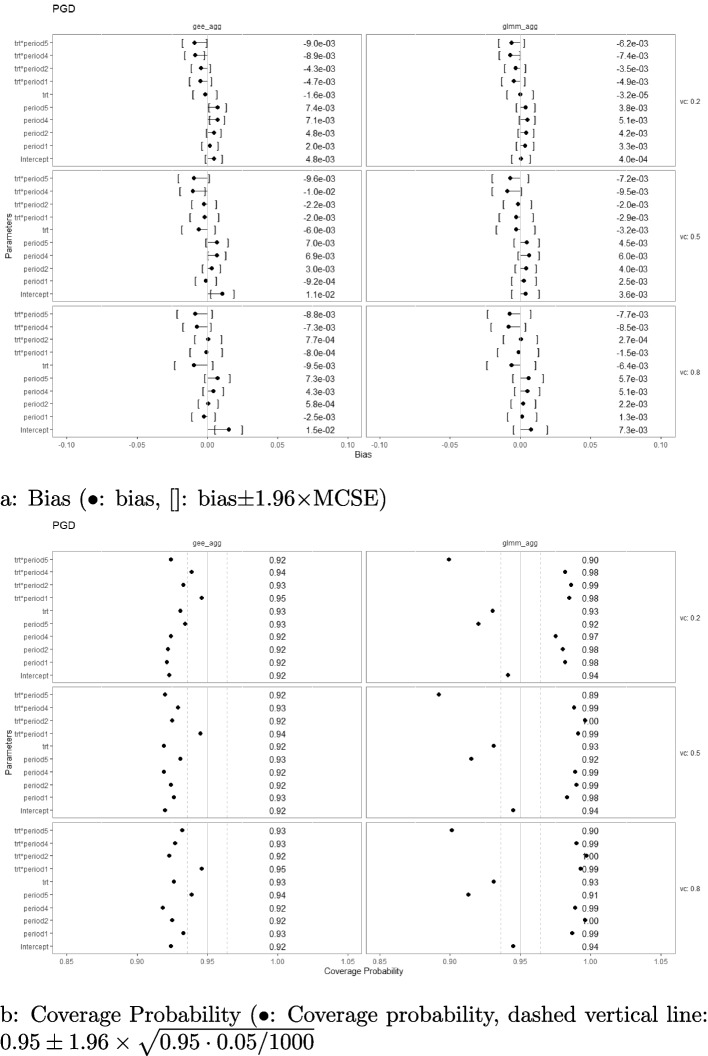
Estimation biases and coverage probabilities of the two approaches (GLMM at aggregated level (glmm_agg), marginal model with GEE at aggregated level (gee_agg)) using the PGD under the settings of $M=48$, $n=50$, linear effect $\delta _j=-0.1(j-3)$, and $\sigma _c^2 \in \{0.2, 0.5, 0.8\}$ with an AR1 correlation structure ($r=0.8$). (vc: variance component, trt*period1-5: treatment-by-period interaction at period 1-5)

**Fig. 9:**
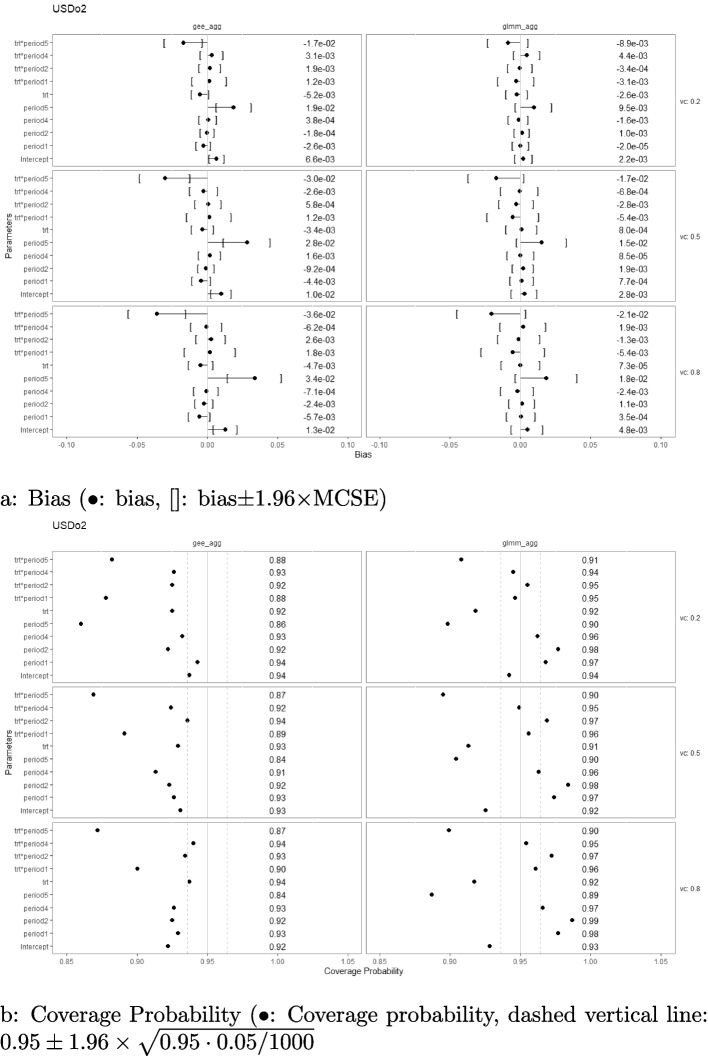
Estimation biases and coverage probabilities of the two approaches (GLMM at aggregated level (glmm_agg), marginal model with GEE at aggregated level (gee_agg)) using the USDo2 under the settings of $M=48$, $n=50$, linear effect $\delta _j=-0.1(j-3)$, and $\sigma _c^2 \in \{0.2, 0.5, 0.8\}$ with an AR1 correlation structure ($r=0.8$). (vc: variance component, trt*period1-5: treatment-by-period interaction at period 1-5)

**Fig. 10:**
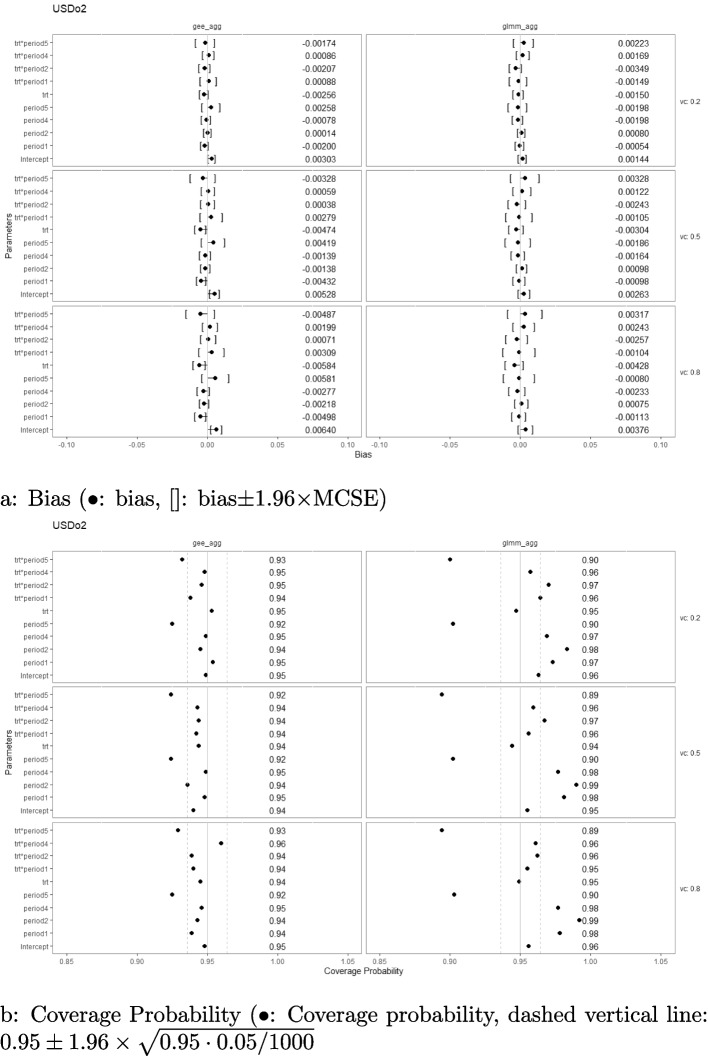
Estimation biases and coverage probabilities of the two approaches (GLMM at aggregated level (glmm_agg), marginal model with GEE at aggregated level (gee_agg)) using the USDo2 under the settings of $M=192$, $n=50$, linear effect $\delta _j=-0.1(j-3)$, and $\sigma _c^2 \in \{0.2, 0.5, 0.8\}$ with an AR1 correlation structure ($r=0.8$). (vc: variance component, trt*period1-5: treatment-by-period interaction at period 1-5)

On the other hand, when the simulated correlation structure is AR1 ($$r=0.8)$$ with $$M=48$$, taking PGD (Fig. 8) and USDo2 (Fig. 9) with linear interaction effect as an example, aggregated-level GEE was not able to estimate certain parameters without bias and consequently had liberal coverages as well. These parameters are period effects at the first and the last period, and treatment-by-period effect at the last period under designs that have extremely unbalanced treatment-control ratios at these periods (namely, USDo1 and USDo2). On the contrary, aggregated-level GLMM did not have issues with the bias of its estimates but suffered from the problem of less stable coverages. Depending on the parameter, the coverages were either liberal or conservative. Nevertheless, as shown in Fig. 10 for the optimal USDo2, the poor performances of GEE diminished when the number of clusters increased while the suboptimal coverages for the parameters remained for the aggregated-level GLMM analysis.

### Type I error and power

The proportion of pure treatment patterns plays an important role in both Type I error and the powers of the four methods as shown in Tables 2 and 3 for the simulation setting with $M=48$, $r=0.8$ for AR1, and a linear interaction effect. First of all, Type I errors of the four methods became more conservative when the proportion of pure treatment patterns decreased from the highest proportion of pure treatment patterns (PGD) to the lowest proportions (USDo2). In this aspect, GEE was more sensitive to the changes in the pure treatment pattern proportions compared to GLMM as the Type I error of GEE was more conservative compared to GLMM among all designs except for the PGD. Nevertheless, no difference was observed between analysis at an aggregated level and individual level for both GEE and GLMM approaches.

**Table 3:** Type I error and power of GLMM and GEE at aggregated level for testing treatment-by-period interactions with AR1 correlation structure ($N_h$ denotes the number of clusters allocated to switch pattern *h*)

Design	$\sigma _c^2$	Cluster allocation						Type I error		Power	
		$N_0$	$N_1$	$N_2$	$N_3$	$N_4$	$N_5$	GLMM Agg	GEE Agg	GLMM Agg	GEE Agg
PGD	0.2	24	0	0	0	0	24	0.077	0.046	0.595	0.406
PGD	0.5	24	0	0	0	0	24	0.078	0.050	0.323	0.181
PGD	0.8	24	0	0	0	0	24	0.076	0.045	0.228	0.128
DSDu	0.2	16	0	0	16	0	16	0.088	0.044	0.510	0.288
DSDu	0.5	16	0	0	16	0	16	0.084	0.037	0.299	0.140
DSDu	0.8	16	0	0	16	0	16	0.076	0.038	0.222	0.101
DSDo	0.2	14	0	0	24	0	10	0.090	0.035	0.431	0.249
DSDo	0.5	14	0	0	24	0	10	0.086	0.040	0.246	0.114
DSDo	0.8	14	0	0	24	0	10	0.007	0.043	0.187	0.086
USDu	0.2	8	8	8	8	8	8	0.073	0.039	0.407	0.195
USDu	0.5	8	8	8	8	8	8	0.083	0.039	0.244	0.101
USDu	0.8	8	8	8	8	8	8	0.078	0.036	0.180	0.074
USDo(1)	0.2	6	9	9	9	9	6	0.090	0.031	0.372	0.157
USDo(1)	0.5	6	9	9	9	9	6	0.081	0.029	0.228	0.085
USDo(1)	0.8	6	9	9	9	9	6	0.082	0.029	0.185	0.068
USDo(2)	0.2	5	10	9	9	10	5	0.085	0.033	0.341	0.144
USDo(2)	0.5	5	10	9	9	10	5	0.083	0.026	0.214	0.068
USDo(2)	0.8	5	10	9	9	10	5	0.071	0.024	0.166	0.061

Furthermore, the proportion of pure treatment patterns also had a substantial effect on the powers of the four methods. The highest power was obtained under the PGD which has the highest proportion of pure treatment patterns. The power of the four methods decreased as the proportion of pure treatment patterns decreased. In addition, GLMM always had higher power compared to GEE, especially when the between-cluster variation is large. Increases in $\sigma _c^2$ also reduced the power of the test within the method and no differences were found between individual and aggregated level analysis for the same method. This phenomenon was less apparent when the simulated correlation structure is AR1. Type I errors and Powers, in general, were worse under AR1 correlation structure for both methods. Considering a MCSE of $\sqrt{0.05\times 0.95 / 1000} \approx 0.007$, Type I errors for the aggregated-level marginal model with GEE were conservative except for PGD while GLMM had a liberal type I error for all designs. The powers of the test were low compared to the power of testing for the overall treatment effect which had more than 50% power in the worst-case scenario. The largest MCSE for the power would be equal to 0.016 when the true power is 50%. Comparing the two analysis methods, GLMM still had higher powers compared to GEE consistently across different designs. However, the power difference between the two did not increase as $\sigma _c^2$ increased.

Similar results were observed when the interaction effect is symmetric and all simulation results for the symmetric interaction effect can be found in Additional files (Additional files 3 and 4).

## Discussion

Simulation results demonstrated that the parallel group design is the best design choice among all studied candidate USDs in terms of the standard error of the estimate for treatment-by-period interactions which consequently results in the highest power for hypothesis testing. Furthermore, GLMM has higher power than the marginal approach with GEE across all studied designs and obtains a better coverage of the true values. Nonetheless, no difference between aggregated and individual-level analysis was found for both GLMM and GEE. Although power comparison between different designs and analysis approaches is not ideal when not all Type I errors are maintained, the differences in the testing powers as demonstrated by the simulation results were substantial and cannot be explained entirely by the difference in Type I errors. Therefore, it is reasonable to conclude that different choices of designs will lead to a systematic difference in the power of hypothesis testing for the treatment-by-period interactions.

The consensus in the current literature is that USD (and its less efficient stepped wedge design variants) is more efficient compared to the PGD in terms of estimating treatment effect under the common settings when the intraclass correlation coefficient is substantial [2, 3, 20, 21]. Our simulation results demonstrate that this does not hold for the treatment-by-period interactions. Parallel group design is by far the most efficient design for treatment-by-period interaction estimations compared to other forms of the USDs. Heuristically, it is not a surprise since none of the candidate designs except for the PGD admits to a balanced treatment allocation within each period. For instance, the uniform USD with an odd number of periods has exactly one period, namely the middle period, that has balanced intervention and control arms. When the number of periods is even, no periods contains balanced treatment allocation. Furthermore, it is known that the power function of the $\chi ^2$-test (of the treatment effect) for a variance component model with the continuous outcome varies inversely with the quantity $\frac{1}{K_0} + \frac{1}{K_1}$ for $K_0$ and $K_1$ the number of clusters exposed to the control and intervention treatment, respectively [22]. Consequently, a balanced allocation ratio is an optimal solution that maximizes the power function. When it comes to testing for the treatment-by-period interaction, it has been shown in the present study that the allocation ratio between the control and intervention arm also plays a similar role on the power such that the parallel group design is the best design choice among all candidates.

In practice, testing for the treatment-by-period interactions is usually conducted after the trial completion with a certain design already chosen [23]. Our simulation results have shown that testing for the treatment-by-period interactions usually won’t have enough power, especially when the correlation structure is not exchangeable. The safest choice when planning a trial when the treatment-by-period interaction is potentially present is to use the PGD. Furthermore, design choices are frequently evaluated for the purpose of estimating the treatment effect alone and does not take into account the treatment-by-period interactions. This has been studied in the literature for randomized trials: the inflation factor for the interaction test to have the same power as the one for the overall treatment effect can be as large as 16 when the interaction is half the size of the overall effect [23]. We recommend also taking into account treatment-by-period interactions when it comes to evaluating the family of USDs.

As for analysis methods, it is preferred to use GLMM rather than GEE. For GEE, estimating the treatment-by-period interactions without bias, when the correlation structure is more complex than exchangeable requires a large number of clusters which may not be feasible in practice. Nevertheless, the difference in power between the GLMM and the marginal model with GEE is mainly due to the differences in the hypothesis testing approaches (i.e., the generalized score test for the marginal model with GEE and Wald’s test for the GLMM). Moreover, the estimator for the covariances of the parameter estimate (i.e., model-based and empirical sandwich estimator) also plays an important role. In GEE, the robust sandwich estimator was used for both the generalized score test and the Wald test while in GLMM either the robust sandwich estimator or the model-based estimator can be adopted. Additional simulation results can be found in the additional file (Additional file 5) where we show the powers of the GLMM and the marginal model with GEE at the aggregated level with AR1 correlation structure ($\sigma _c^2=0.8, r=0.8$) under USDo2 using different hypothesis testing approaches in combination with both model-based and robust sandwich standard error estimators. In line with previous study [24], the robust Wald test was more liberal and had higher power than the generalized score test for the marginal model with GEE. It had similar power compared to the power of the $\chi ^2$ test used in GLMM with the model-based estimator. Meanwhile, using the robust estimator for GLMM parameter estimates in place of the model-based estimator brought the power of the test down to the same level as the power of the generalized score test. Some authors have performed comparative work in this area [24, 25], but it is largely a work in progress which falls outside the scope of our presented study.

One of the limitations of the presented study is that we only considered a handful of settings and models. For instance, we did not consider the time-on-treatment effects [6] which might be considered as a three-way interaction between treatment, period, and cluster. Therefore, it is important to study the problem of treatment-by-period interaction from a more theoretical perspective and consider different models under the family of USDs. Another limitation is that we only consider binary outcomes. For continuous and time-to-event outcomes, the impact of treatment-by-period interaction is yet to be investigated.

## Conclusions

In the present study, we compared four analysis methods for estimating and testing the treatment-by-period interactions under various unidirectional switch design choices. Via Monte Carlo simulation, it has been found that the parallel group design is the most efficient in terms of estimating and testing the treatment-by-period interactions. Whilst no substantial difference is observed between analysis at an aggregated level and individual level, GLMM has higher efficiencies and better point estimates compared to the marginal model with GEE under different designs. Its coverages were worse than the marginal model with GEE when the correlation structure is AR1 even when the number of clusters is as high as 192.

## Supplementary Information

**Additional file 1.** Simulation results (Point estimates, Coverage probabilities, Type I error, and Power) for linear interaction effects with $M=48$.

**Additional file 2.** Simulation results (Point estimates, Coverage probabilities, Type I error, and Power) for linear interaction effects with $M=192$.

**Additional file 3.** Simulation results (Point estimates, Coverage probabilities, Type I error, and Power) for symmetric interaction effects with $M=48$.

**Additional file 4.** Simulation results (Point estimates, Coverage probabilities, Type I error, and Power) for symmetric interaction effects with $M=192$.

**Additional file 5.** Simulation results (Power) for linear interaction effects with $M=48$ of GLMM and GEE at aggregated level with AR1 correlation structure $\sigma_{c}^{2}=0.8,\ r=0.8$ under USDo2 with different hypothesis testing methods and with different estimators for the standard error of the parameter estimates.

## Data Availability

The datasets generated and/or analysed during the current study are available from the corresponding author on reasonable request. All SAS and R codes used in this study can be found at https://github.com/jujae/USD-interaction.

## Abbreviations

USD: Unidirectional switch design
GLMM: Generalized linear mixed model
GEE: Generalized estimating equations
PGD: Parallel group design
SWD: Stepped wedge design
DSD: Delayed start design
AR1: Autoregressive order 1

## Footnotes

1 Here we picked $M=48$ because we choose the number of clusters to be divisible by the number of patterns $T+1 = 6$ and to make the differences between USDu, USDo1, and USDo2 substantial
